# Reciprocity models revisited: intention factors and reference values

**DOI:** 10.1007/s00182-024-00898-z

**Published:** 2024-05-21

**Authors:** Janna Hinz, Andreas Nicklisch, Mey-Ling Sommer

**Affiliations:** 1 Department of Economics, University of Hamburg, von-Melle-Park 5, 20146 Hamburg, Germany; 2 Center for Economic Policy Research, University of Applied Sciences of the Grisons, and Research Unit “Needs based Justice and Distribution Procedures”, Comercialstr. 20, 7000 Chur, Switzerland; 3 Faculty of Economics und Social Sciences, Helmut Schmidt University, Holstenhofweg 85, 22043 Hamburg, Germany

**Keywords:** Experiments, Intentions, Mini-ultimatum game, Reference value, Reciprocity models, C52, C72, C91

## Abstract

We present a test of the two most established reciprocity models, an intention factor model and a reference value model. We test characteristic elements of each model in a series of twelve mini-ultimatum games. Results from online experiments show major differences between actual behavior and predictions of both models: the distance of actual offers to the proposed reference value provides a poor measure for the kindness of offers, while a comparison of offers with extreme offers as suggested by the intention factor model makes offers indiscriminable in richer settings. We discuss possible combinations of both models better describing our observations.

## Introduction

Reciprocity is one fundamental cornerstone of human behavior, and an integral element for other-regarding preferences. The importance of reciprocal behavior for human interactions has been stressed by a large body of economic literature (e.g., Cox and Deck 2003, Andreoni et al. (2003); Falk et al. (2003); Gächter and Thöni (2007)).^1^ Consequently, behavioral economists have been striving to explain how people digress from self-interested behavior to reward kind actions and punish unkind actions of their opponents. Modeling reciprocity, however, has turned out to be a very complex endeavor. The specific formulation of reciprocal preferences follows predominantly two distinct ways: Rabin (1993) and Dufwenberg and Kirchsteiger (2004) focus on an comparison between a “suggested” payoff and a reference value. In turn, Falk and Fischbacher (2006) rely on a combination of the inequality of the “suggested” payoffs and an intention factor measuring how deliberate the “suggestion” is to capture underlying motivations. Despite the ambiguity of both approaches, their scientific impact is enormous^2^ leading subsequent studies to rely on one or the other theory (e.g., Stanca et al. (2009), Ambrus and Pathak 2012).

In this current study we test key characteristic features of both approaches in a number of mini-ultimatum games similar to the one used in the tradition of Bolton and Zwick (1995) and Falk et al. (2003). Particularly, we focus on two key differences between the two approaches: firstly, the reference value approach measures the extent of (un)kindness according to the distance of the specific offer to the reference value, while the intention factor approach measures the unkindness by the inequity of the specific offer. Secondly, although both models take extreme outcomes into considerations for assessing (un)kindness, the reference value appears more robust against outliers: outliers along other alternative outcomes of the game are converted into a reference value, whereas the intention factor approach measures the intention by a pairwise comparison between an extreme outcome and the specific offer. We show that both approaches have advantages and disadvantages when explaining actual decisions so that a combination of both approaches seems to provide a good description of behavior.

The general idea of reciprocal preferences is perhaps best summarized by the Latin principle ‘quid pro quo.’ The overarching non-parametric model by Cox et al. (2008) formalizes these words in the following way: suppose a proposer (player *P*) has a number of alternatives from which she can choose one. Her choice has consequences in terms of payoffs not only for herself, but also for the responder (player *R*). Among the alternatives player *P* can choose from, player *R* considers *P*’s choice *blue* to be more generous than *red* if *blue* yields a higher payoff to *R* than the choice of *red*, while *P*’s gain from choosing *blue* and not *red* is at most as large as *R*’s gain from choosing *blue* and not *red*. *R* may or may not accept the proposed alternative. With *R* holding reciprocal preferences, the likelihood of *R*’s approval for a suggested alternative increases, the more generous *P*’s choice is: *R* gains *immaterial* utility from *P*’s material payoffs if *P* behaves generously, whereas *R*’s immaterial utility can even be negative, if *P* chooses a mean alternative. Consequently, *R* may want to punish *P* if *P* behaved unkindly, for instance by destroying all payoffs altogether.

Contemporary reciprocity concepts translate the general idea of quid pro quo into well-defined closed preference models. We refer to the first approach as the “reference value model,” first formalized by Rabin (1993).^3^ In his belief-dependent model, reciprocity is analyzed for two-player, normal-form games. Dufwenberg and Kirchsteiger (2004; herafter D &K) extended Rabin’s model of belief-dependent preferences to extensive *n*-player games. In both models, *R* ranks *P*’s alternatives from the one yielding the lowest payoff for *R* to the highest payoff for *R*. Half way between *R*’s lowest and highest payoff lies *R*’s *equitable payoff* dividing *P*’s alternatives into unkind ones below the equitable payoff and kind ones above (hereafter, we denote the equitable payoff as the reference value). *P*’s *(un)kindness towards* *R* increases in the difference between the payoff corresponding to *P*’s choice and the reference value. We would like to stress that the reference value model measures the action’s kindness by a “global assessment.” That is, the midpoint of the entire set of alternatives in the game determines the reference value which, in turn, determines the kindness of a specific offer.

We test the predictive success of the reference value model according to two characteristics. Firstly, we check for the continuity of the reference value: we analyze whether the likelihood that *R* accepts an alternative increases in the distance between the equitable payoff and the payoff “normally resulting” from *P*’s chosen action.^4^ Secondly, we test for the predictive success of the reference value: varying the game, but keeping the reference value and the distance from the reference value constant, we analyze whether the likelihood that *R* responds unkindly (i.e., she does not accept) remains constant.^5^

The second class of reciprocity models, “intention factor models,” contrasts the reference value models in two ways: they decompose *P*’s (un)kindness towards *R* into the product of an intention term and an outcome term (e.g., Falk and Fischbacher (2006), hereafter F &F). The first term determines whether *R* perceives *P*’s action as intended or not, the second term determines the severeness of *P*’s perceived (un)kindness. F &F place in *R*’s immaterial partial utility from reciprocity prior importance on the difference of payoffs between *P* and *R* within an option before the difference to the other possible payoffs for *R* is considered. As mentioned earlier, the intention factor model assesses the action’s kindness in a pairwise comparison with extreme outcomes of the game. This may yield a problem for this approach: one kind alternative can turn all other alternatives inevitable into fully intentional unkind alternatives. We will show the consequences of this feature for predictions in games with several alternatives in Section 3.

The crucial importance of intentions for reciprocal behavior has been shown elsewhere (e.g. Falk et al. (2008)). We test the predictive success of intentions by checking for the continuity of the intention factor: we analyze whether the likelihood that *R* responds unkindly increases, if the intention factor increases, keeping the inequity of *P* and *R*’s payoffs constant. Secondly, we test for consistency of the intention factor: varying the game, but keeping the intention factor and the inequity of payoffs constant, we analyze whether the likelihood that *R* responds unkindly remains constant. In other words, we test whether an assessment of kindness relying on the pairwise comparison describes *P*’s perceived kindness properly.

For our purpose, we propose a series of twelve mini-ultimatum games. Some of them offer *P* two alternatives to choose from, some of them offer four alternatives. All of them allow *R* to reject a proposed alternative and forgo her own income for the sake of punishing *P*. The games are designed such that they allow us to assess the predictive success of reference value models and intention factor models. We retrieve our data in online experiments with almost 500 participants. As such, our analysis follows Sobel (2005) criticism that existing reciprocity models seem to be fitting for specific situations, but lack a clear characterization of this very situation. Along the same line of arguments, there are some other studies discussing and testing the predictive success of reciprocity models. Firstly, they provide evidence on the importance of intentions for reciprocation: if there is no alternative but to behave unkindly, subjects reciprocate less severely (Falk et al. 2003); the same holds true if an action is taken that is not unambiguously kind, but selfish to some degree (Stanca et al. 2009). Secondly, Dhaene and Bouckaert (2010) elicit first and second order beliefs of participants in a sequential prisoners’ dilemma and a mini-ultimatum game. They show that beliefs and behavior, particularly of second movers, are very consistent with D &K’s reciprocity model. Furthermore, Pelligra (2011) varies systematically the outside options in a trust game, where the first mover’s trusting is either kind or unkind for the second mover. Contrasting the theoretical predictions of the D &K model, the trustworthiness of the second mover remains constant across treatment conditions suggesting that other motives dominate behavior in this setting (cf., Pelligra (2011)). Finally, Nicklisch and Wolff (2012) test an overall characteristic of reference value models and intention factor models: if punishment is sufficiently cheap, reciprocation is modeled as an “all-or-nothing” decision. That is, if *P* behaves kindly (unkindly), *R* maximizes her utility by choosing the most kind (unkind) response possible. By means of a modified ultimatum game, the authors show that decisions for a majority of participants in a laboratory experiment do not follow this assumption.

Along this partly pessimistic assessment of contemporary reciprocity models, our data shows important shortcomings for both models when predicting behavior. Particularly, the continuity of the reference value model fails to characterize actual behavior: increasing the distance between the reference value and the payoff of the actual offer does not necessarily correspond with increasing rejection rates. Moreover, variation of the game yields differences in the rejection rates although the reference value and the distance from the reference value remain constant. We conclude from those findings that the distance to the reference value serves as a poor descriptor for the extent of (un)kindness. On the other hand, experimental results for simple games with two alternatives are nicely predicted by the intention factor model. The likelihood of rejection increases for increasing intention factors. However, there is little consistency between predictions and decisions in the richer games with four alternatives. We conclude from this that the pairwise comparison of alternatives does not characterize behavior adequately, and suggest a combination of both approaches. This combination includes a global assessment for the intention of a choice and the inequity of an alternative for the extent of (un)kindness.

The remainder of this article is organized as follows: The following section (re)acquaints with both reciprocity models to be tested with an emphasis on the element we scrutinize. In Section 3 we introduce our experimental design and procedure. Section 4 presents results. In Section 5 we discuss our findings and suggest potential developments for reciprocity models reflecting our results. Section 6 concludes.

## Reciprocity based utility

Let us consider a two-player extensive form game with two stages and common knowledge about the game and decisions in previous stages.^6^ Formally, suppose player *P* and player *R* be proposer and responder in an ultimatum-like game. *P* chooses an action $a_p$ from her set of alternatives $A_p$. Suppose $a_p$ affects *P*’s and *R*’s payoffs ($\pi _p$ and $\pi _r$, respectively). *R* observes *P*’s action. Then *i*’s reciprocity based utility function with $i,j \in \{P,R\}$ has the following structure:

1 $$\begin{aligned} U_{i} = \pi _{i}(a_i,a_j)+\psi _{i}\kappa _{ji}(a_j,b'_{ij},b''_{iji})\lambda _{ij}(a_i,b'_{ij},b''_{iji}) \end{aligned}$$

Utility consists of a material payoff $\pi _i$ and an immaterial utility component. The material part of $U_{i}$ refers to the payoff assigned to the outcome of a specific choice. The immaterial part of utility is initiated with an individual sensitivity parameter to reciprocal concerns, $\psi _{i}$. If $\psi _{i}=0$, then utility will equal material payoff as suggested by narrow self-interest. $\kappa _{ji}(.)$ denotes *i*’s perceived (un)kindness of *j*’s action, and $\lambda _{ij}(.)$ is the (un)kindness of *i*’s response. The latter two depend on *i*’s (first-order) expectations concerning *j*’s behavior in the consecutive game ($b'_{ij}$), and *i*’s (second-order) expectations concerning *j*’s beliefs of *i*’s beliefs of the consecutive game ($b''_{iji}$).

Depending on the first- and second-order beliefs of players, reciprocity models can accommodate generally a very large range of behavior. For instance, a very reciprocal responder may expect to receive a very low offer, perceiving a large offer to be mean. While she assumes that the proposer expects her to accept the offer, she may decide to reject the large offer yielding substantial immaterial utility resulting from the mean rejection of a mean offer. In turn, a very reciprocal proposer^7^ may consider the rejection of offers to be mean. Anticipating that responders consider large offers as mean, the proposer casts a large (mean) offer facing the (anticipated) mean rejection. Consequently, both proposer and responder yield higher utility by a rejection of a large offer than through the acceptance of a small offer.^8^

Whereas reciprocity allows generally for “masochistic” equilibria that maximize the immaterial utility component of mutual mean actions (see our last footnote), we rule those equilibria out as non-plausible in our dictator games. That is, we assume that proposers do not submit offers for which they seek a rejection in order to maximize their immaterial utility.^9^ Notice that D&K’s approach allows in principle for those equilibria based on mutual meanness. Hence, we do not test D &K’s full model but dismiss some of its equilibria on plausibility grounds.

Thus, we assume in the following that if a proposer casts an offer, she expects its acceptance, and she expects the responder to believe that she aims at the acceptance of the offer. Formally, $b'_{PR}=$“*acceptance*” and $b''_{PRP}=$“$b'_{RP}=acceptance$”, while $b'_{RP}=$“*acceptance*” and $b''_{RPR}=$“$b'_{PR}=acceptance$”. As a consequence, the terms for *i*’s perceived (un)kindness of *j*’s action and the (un)kindness of *i*’s response simplify to $\kappa _{ji}(a_j)$ and $\lambda _{ij}(a_i)$, since proposer’s and responder’s first order and second order beliefs assume the acceptance of the offer. That is, “the normally resulting way” of the ultimatum game assumes the acceptance of the proposed offer. Whether actual behavior $a_R$ deviates from the expected way, is in both models a matter of the immaterial partial utility components from reciprocity.

In the next sections, we briefly present the key components of both reference value models and intention factor models. Both models provide a solution concept that require an updating of players’ beliefs as the play unfolds.^10^ We will pay special attention to the differences in the updating between both approaches, and formulate hypotheses based on the theoretical analysis.

### Sequential reciprocity according to Dufwenberg & Kirchsteiger

With respect to the reference value model by Dufwenberg & Kirchsteiger, *R*’s immaterial partial utility from reciprocity equals the product of *P*’s (un)kindness towards *R*, $\kappa _{PR}(a_P)$, and *R*’s (un)kindness towards *P* by deviating from “the normally resulting way”, $\lambda _{RP}(a_R)$.

*R*’s reference value separating *P*’s actions into kind and unkind actions is the value half way between the lowest and highest payoff available at the time when *P* makes her decision. Formally, *R*’s equitable payoff $\pi _{R}^{e_{P}}$ (i.e., *R*’s average payoff following *P*’s action) is:

2 $$\begin{aligned} \pi _{R}^{e_{P}}= 0.5\max (\pi '_{R})+0.5\min (\pi '_{R}), \end{aligned}$$

where $\pi '_{R}$ is the set of payoffs induced by *P*’s efficient strategies. In turn, not considered are *P*’s inefficient strategies, that is, strategies for which one finds a Pareto improvement – in terms of *R*’s and *P*’s payoffs – among *P*’s strategies for any strategy choice of *R* (compare D &K, pp. 275–276).

*P*’s (un)kindness towards *R* in D &K’s approach is evaluated according to the difference between the payoff resulting from accepting *P*’s offer and the reference value:

3 $$\begin{aligned} \kappa _{PR}^{D \& K}(a_P) = \pi _{R}(a_P)- \pi _{R}^{e_{P}} \end{aligned}$$

After *R* observes *P*’s move, it is on her to respond, again influencing the monetary outcomes for both players. That is, ranking *R*’s efficient alternatives from the one yielding the lowest to the highest payoff for *P*, the midpoint of the ranking determines *R*’s reference value for the (un)kindness of her response. In other words, *R*’s (un)kindness towards *P* is measured according to the distance between *P*’s actual payoff to *P*’s equitable payoff. It is important to stress that *P*’s equitable payoff $\pi _{P}^{e_{R}}$ in the ultimatum game is the payoff resulting from accepting the offer: recall that *P*’s equitable payoff averages the maximum and the minimum in the set of payoffs induced by *R*’s efficient strategies. Since rejections are inefficient, *R*’s only efficient strategy equals the acceptance of the offer. Therefore, it follows:

4 $$\begin{aligned} \lambda _{RP}^{D \& K}(a_R) = \pi _{P}(a_R)- \pi _{P}(acceptance) \end{aligned}$$

D &K’s explicit quantification of (un)kindness with the equitable payoff as a reference value allows us to test the predictive success of their model:

Hyp_D&K_: *Keeping the offer constant, but decreasing the distance to the equitable payoff across the proposer’s actions implies non-increasing rejection rates for those actions, while keeping the distance constant implies a constant rejection rate*.

### Intention-based reciprocity according to Falk & Fischbacher

Within the context of our simple mini-ultimatum games, one can show for Falk & Fischbacher’s approach that $\lambda _{RP}^{F \& F}(a_R)=\lambda _{RP}^{D \& K}(a_R)$: according to F &F, the kindness of *R*’s reciprocation is measured with respect to the degree by which *R* alters *P*’s actual from his expected payoff. Since *R* expects *P* to propose her an offer for which *P* seeks *R*’s acceptance in our mini-ultimatum games, *R* alters *P*’s payoff by rejecting the offer implying a negative reciprocation.

In turn, the difference between the two approaches is settled in the specific form of $\kappa _{PR}^{F \& F}(a_P)$. F &F separate the perceived (un)kindness into two terms, the outcome term $\Delta _P(a_P)$ and the intention factor $\vartheta _{P}^{F \& F}(a_P)$. The outcome term is formalized such that the evaluation of kindness is based on the inequity between proposer’s and responder’s payoffs at a specific end node assuming that the responder chooses her efficient strategy:

5 $$\begin{aligned} \Delta _{P}(a_P)= \pi _{R}-\pi _{P} \end{aligned}$$

Again, within the context of our mini-ultimatum games, we can simply insert the payoffs following accepted offers into Eq. (5).

To derive perceived kindness, the outcome term is multiplied with the intention term, where reciprocal concerns come into play. Here, F &F distinguish between five payoff constellations from which different intentions are derived. More specifically, the intention factor accounts for the proposer’s intentional and unintentional choices depending on how she could have altered payoff constellation with regard to her own in combination with the responder’s payoff:

6 $$\vartheta _{P}^{{F\& F}} (a_{P} ) = \left\{ {\begin{array}{*{20}c} {1\;\;\;\;\;\;\;\;\;\;\;\;\;\;\;\;\;\;\;\;\;\;\;\;\;\;\;\;} & {{\text{if }}\pi _{R}^{0} \ge \pi _{P}^{0} {\text{ and }}\exists \tilde{\pi }_{R} \in \tilde{\Pi }_{R} :\tilde{\pi }_{R} < \pi _{R}^{0} ,\;\;\;\;\;\;\;\;\;\;\;\;\;\;\;\;\;\;\;} \\ {{\epsilon}_{R} \;\;\;\;\;\;\;\;\;\;\;\;\;\;\;\;\;\;\;\;\;\;\;\;\;\;\;} & {{\text{if }}\pi _{R}^{0} \ge \pi _{P}^{0} {\text{ and }}\forall \tilde{\pi }_{R} \in \tilde{\Pi }_{R} :\tilde{\pi }_{R} \ge \pi _{R}^{0} ,\;\;\;\;\;\;\;\;\;\;\;\;\;\;\;\;\;\;\;} \\ {1\;\;\;\;\;\;\;\;\;\;\;\;\;\;\;\;\;\;\;\;\;\;\;\;\;\;\;\;} & {{\text{if }}\pi _{R}^{0} < \pi _{P}^{0} {\text{ and }}\exists \tilde{\pi }_{R} \in \tilde{\Pi }_{R} :\tilde{\pi }_{R} > \pi _{R}^{0} {\text{ and }}\tilde{\pi }_{R} \le \tilde{\pi }_{P} } \\ {\max \left( {1 - \frac{{\tilde{\pi }_{R} - \tilde{\pi }_{P} }}{{\pi _{P}^{0} - \pi _{R}^{0} }},{\epsilon}_{R} } \right)} & {{\text{if }}\pi _{R}^{0} < \pi _{P}^{0} {\text{ and }}\exists \tilde{\pi }_{R} \in \tilde{\Pi }_{R} :\tilde{\pi }_{R} > \pi _{R}^{0} {\text{ and }}\tilde{\pi }_{R} > \tilde{\pi }_{P} } \\ {{\epsilon}_{R} \;\;\;\;\;\;\;\;\;\;\;\;\;\;\;\;\;\;\;\;\;\;\;\;\;\;} & {{\text{if }}\pi _{R}^{0} < \pi _{P}^{0} {\text{ and }}\forall \tilde{\pi }_{R} \in \tilde{\Pi }_{R} :\tilde{\pi }_{R} \le \pi _{R}^{0} ,\;\;\;\;\;\;\;\;\;\;\;\;\;\;\;\;\;\;} \\ \end{array} } \right.$$

where $\pi _{R}^{0}$, $\pi _{P}^{0}$ are payoffs resulting from accepting the specific offer in our design. Let ${\tilde{\Pi }}_R$ be the set of payoffs resulting from the acceptance of an alternative offer (but not the specific offer), ${\tilde{\pi }}_{R}$ be one element in ${\tilde{\Pi }}_R$, and $\epsilon _{R}$ be an individual parameter with $0\le \epsilon _R \le 1$. This parameter is denoted as the pure outcome concern parameter. That is, $\epsilon _{R}$ measures the responder’s unease with the inequity between proposer’s and responder’s payoff, although the proposer has no option to avoid the kind or mean offer.

The first two cases of (6) refer to intentions of the proposer’s actions favoring the responder money-wise: in the first one, the proposer offers the responder not the smallest payoff possible although it is higher than his own one. This case is considered as fully intentional. In the second case, the proposer offers the responder a higher payoff than her own one, but has no chance to avoid this. In this case, the outcome is nice, but the proposer does not act intentionally so that the intention factor is reduced. The last three cases refer to negative intentions: the final case mirrors the second case into the negative domain; the proposer offers the responder a smaller payoff than her own one, but has no chance to avoid this. In this case, the outcome is mean, but the proposer does not act intentionally so that the intention factor is reduced, whereas the third and fourth case refer to intentionally mean choices. In the fourth one, the proposer offers the responder a smaller payoff than a possible alternative, but the offer is “somehow understandable” in the sense that the alternative yields less for the proposer than for responder. Therefore, the proposer’s intention is discounted according to the proposer’s relative disadvantage under the alternative. In contrast, in the third case, the proposer’s unkindness is fully intentional, since there is a better alternative for the responder, which does not yield a lower payoff for the proposer than for responder.

Putting both terms together, $\kappa _{PR}^{F \& F}(a_P)$ consists of the outcome term multiplied with the intention factor:

7 $$\begin{aligned} \kappa _{PR}^{F \& F}(a_P) = \vartheta _{P}^{F \& F}(a_P)\Delta _{P}(a_P) \end{aligned}$$

In other words, to derive kindness in the intention factor model, intentions are “charged” by the difference between the proposer’s and responder’s monetary payoff, namely the outcome term. Inequity in favor of the responder increases the severity of the proposer’s kindness towards the responder, while disadvantageous inequity for the responder increases the severity of the proposer’s unkindness towards the responder. This means that the likelihood for the responder to behave unkindly (kindly) increases the more her normally resulting payoff falls below (surpasses) the proposer’s payoff, given that the proposer chooses fully intentionally. While F &F’s model elegantly combines inequality aversion with reciprocal motivations, the formalization of the intention factor hosts strong, testable assumptions. Specifically, F &F’s explicit quantification of the outcome term allows us to test the predictive success of their model:

Hyp_F&F_: *Decreasing the intention factor while keeping the offer constant across the proposer’s actions implies non-increasing rejection rates for those actions, while keeping the intention factor constant implies a constant rejection rate.*

## The games

### Design

To test both reciprocity models we design a series of twelve systematically varying mini-ultimatum games $\Gamma _1$ to $\Gamma _{12}$ similar to the design by Falk et al. (2003). The proposer receives throughout all games an endowment of at most 10 Talers.^11^ In games $\Gamma _1$ to $\Gamma _{7}$, the proposer decides among two alternatives (green, red), in $\Gamma _8$ to $\Gamma _{12}$ she decides among four alternatives on how to split the 10 Talers between the responder and herself (green, red, yellow, blue). The responder can either accept or reject the proposer’s offer; in the former case, both parties reap their designated payoff, in the latter case both players receive zero Talers. In all twelve games one allocation is held constant at (8, 2), while the remaining allocations differ depending on the purpose of each game. Table 1 lists all payoff allocations for the whole series of games.

**Table 1:** Payoff alternatives in $\Gamma _1$ to $\Gamma _{12}$ along the values of $\kappa _{PR}^{D \& K}(8,2)$ and $\vartheta _{P}^{F \& F}(8,2)$ for the offer (8, 2) according to the corresponding theories

	Green	Red	Yellow	Blue	$\kappa _{PR}^{D \& K}(8,2)$	$\vartheta _{P}^{F \& F}(8,2)$
$\Gamma _1$	(8, 2)	(8, 2)			0	$\epsilon _{R}$
$\Gamma _2$	(8, 2)	(5, 5)			$-1.5$	1
$\Gamma _3$	(8, 2)	(9, 1)			0.5	$\epsilon _{R}$
$\Gamma _4$	(8, 2)	(7, 3)			$-0.5$	1
$\Gamma _5$	(8, 2)	(4, 3)			$-0.5$	1
$\Gamma _6$	(8, 2)	(3, 7)			$-2.5$	$\max (\frac{2}{6},\epsilon _{R})$
$\Gamma _7$	(8, 2)	(3, 4)			$-1.0$	$\max (\frac{5}{6},\epsilon _{R})$
$\Gamma _8$	(10, 0)	(9, 1)	(8, 2)	(5, 5)	$-0.5$	1
$\Gamma _9$	(9, 1)	(7, 3)	(8, 2)	(5, 5)	$-1.0$	1
$\Gamma _{10}$	(7, 3)	(6, 4)	(8, 2)	(5, 5)	$-1.5$	1
$\Gamma _{11}$	(9, 1)	(10, 0)	(8, 2)	(6, 4)	0	1
$\Gamma _{12}$	(9, 1)	(7, 3)	(8, 2)	(6, 4)	$-0.5$	1

Of course, non-reciprocal social preferences (e.g., inequity aversion) do not predict any difference concerning the likelihood to reject (8, 2) across games (e.g., Fehr and Schmidt (1999), and Bolton and Zwick (1995)), but also models with a scope beyond the pure outcome of interactions (e.g., Levine (1998); see the Appendix for further discussions). This changes substantially once we consider reciprocity. For our experiment, $\Gamma _1$ and $\Gamma _2$ serve as our baseline games. They provide benchmarks for our analysis in the sense that $\Gamma _1$ gives some indications for the responder’s pure outcome concern (i.e., $\epsilon _{R}$). That is, there is no intention involved in the offer green in $\Gamma _1$, since the proposer has no alternative given that green equals red. In other words, rejecting in $\Gamma _1$ shows that the responder is ready to forgo 2 Talers, because she is so inequity averse that she does not want the proposer to gain 6 Talers more than her. Thus, rejections in $\Gamma _1$ indicate strong outcome concerns. On the other hand, $\Gamma _2$ shows us the response to a fully intentional, very unkind offer according to the two reciprocity models. That is, $\Gamma _2$ introduces a large distance between 2 and the equitable payoff ($\pi _{P}^{e_{R}}=3.5$ in $\Gamma _2$), while the offer is made with full intentions according to F &F.

With $\Gamma _3$ to $\Gamma _7$ we address both models in the context of simple games (i.e., games with two alternatives). According to F &F, $\Gamma _3$ depicts the fifth case of the intention factor resulting in an intention factor equal to the individual outcome concern parameter $\epsilon _{R}$. As the outcome term is the same as in $\Gamma _1$, with reference to Hyp_F&F_ the rejection rate of green is predicted to be equal to the rejection rate of $\Gamma _1$. Particularly, responders rejecting (8, 2) in $\Gamma _1$ are expected to reject (8, 2) in $\Gamma _3$ and vice versa.

$\Gamma _4$ and $\Gamma _5$ both depict a fully intentional decision context, corresponding to the third case of the intention factor. Thus, with reference to Hyp_F&F_ we predict equal rejection rates of green for both games such that responders reject or accept (8, 2) in both $\Gamma _4$ and $\Gamma _5$. $\Gamma _6$ and $\Gamma _7$ reverse payoffs of the two previous games in the alternative red, resulting in a decision context characterized in the fourth case of the intention factor. Due to this structure, F &F’s model predicts slightly smaller rejection rates of green in $\Gamma _7$ (i.e., $\vartheta _{P}^{F \& F}(8,2)$ is at least $\frac{5}{6}$ in this game), whereas the rejection rate in $\Gamma _6$ is predicted to be lower than in $\Gamma _4$, $\Gamma _5$ and $\Gamma _7$ – at least for those subjects whose choice indicated low inequity concerns (i.e., $\epsilon _R$) by accepting green and red in $\Gamma _1$. Based on Hyp_F&F_, this implies on an individual basis that responders rejecting (8, 2) in $\Gamma _6$ are expected to reject (8, 2) in $\Gamma _4$, $\Gamma _5$ and $\Gamma _7$, while responders rejecting (8, 2) in $\Gamma _7$ are expected to reject (8, 2) in $\Gamma _4$ and $\Gamma _5$.

Let us now turn to the alternative theory: according to D &K, $\Gamma _3$ has a positive distance to the equitable payoff implying that rejections decrease utility. Therefore, responders do not reject (8, 2) in $\Gamma _3$. $\Gamma _4$ and $\Gamma _5$ both depict the same distance to the equitable payoff suggesting equal rejection rates of green for both games. In line with the predictions for F &F, responders rejecting (8, 2) in $\Gamma _4$ are expected to reject (8, 2) in $\Gamma _5$ and vice versa. However, D &K’s predictions for $\Gamma _6$ and $\Gamma _7$ change substantially in comparison to F &F. $\Gamma _6$ introduces the most extreme distance to the equitable payoff within our sample of games, whereas $\Gamma _7$ introduces a smaller distance to the equitable payoff (though larger than in $\Gamma _4$ and $\Gamma _5$). It follows that $\Gamma _6$ has the largest rejection rate for (8, 2), followed by $\Gamma _2$, $\Gamma _7$, and $\Gamma _4$ and $\Gamma _5$. This implies on an individual basis that responders rejecting (8, 2) in $\Gamma _4$ and $\Gamma _5$ are expected to reject (8, 2) in $\Gamma _2$, $\Gamma _6$ and $\Gamma _7$, while responders rejecting (8, 2) in $\Gamma _7$ ($\Gamma _2$) are expected to reject (8, 2) in $\Gamma _2$ and $\Gamma _6$ ($\Gamma _6$).

$\Gamma _8$ through $\Gamma _{12}$ are designed to test the models in a richer context (i.e., games with more than two alternatives). Notice that the predictions according to F &F are constant as they depict the third case of the intention factor resulting in an intention factor equal to 1 for all games $\Gamma _8$ to $\Gamma _{12}$. In all games, the proposer’s choice of yellow is fully intentional due to the pairwise comparison between (8, 2) and (5, 5) ($\Gamma _{8}$ to $\Gamma _{10}$), or between (8, 2) and (6, 4) ($\Gamma _{11}$ and $\Gamma _{12}$). In other words, the other alternatives except (5, 5) ((6, 4)) are irrelevant for determining the intention of choosing (8, 2). Hence according to Hyp_F&F_ the rejection rate for (8, 2) is equal across all five games, such that responders either have to reject or accept (8, 2) in all games $\Gamma _2$, $\Gamma _4$, $\Gamma _5$ and $\Gamma _8$ to $\Gamma _{12}$.

Following D &K, $\Gamma _8$ through $\Gamma _{10}$ represent a sequence of rising reference values implying increasing rejection rates for yellow according to Hyp_D&K_: reference values increase from 2.5 in $\Gamma _8$ to 3 in $\Gamma _9$ to 3.5 in $\Gamma _{10}$ suggesting that rejection rates are expected to increase from $\Gamma _8$ through $\Gamma _{10}$. In turn, responders rejecting 8, 2 in $\Gamma _8$ are expected to reject 8, 2 in $\Gamma _9$ and $\Gamma _{10}$, while responders rejecting 8, 2 in $\Gamma _9$ are expected to reject 8, 2 in $\Gamma _{10}$.

$\Gamma _{11}$ and $\Gamma _{12}$ substitute the option blue allowing us some interesting comparisons within the richer games and across all games. According to Hyp_D&K_, the rejection rate of $\Gamma _{12}$ is predicted to be equal to that of $\Gamma _4$, $\Gamma _5$ and $\Gamma _8$ (likewise, the rejection rate of $\Gamma _{7}$ is predicted to be equal to that of $\Gamma _9$, the rate for $\Gamma _2$ to be equal the rate of $\Gamma _{10}$). Responders rejecting (8, 2) in $\Gamma _4$, $\Gamma _5$, $\Gamma _8$ and $\Gamma _{12}$ are expected to reject (8, 2) in $\Gamma _7$, $\Gamma _9$, $\Gamma _2$, $\Gamma _{10}$ and $\Gamma _6$, as the distance to the equitable payoff is smaller in the former than in the latter games according to Hyp_D&K_. Likewise, substituting (5, 5) in $\Gamma _{8}$ against (6, 4) in $\Gamma _{11}$ changes the character of (8, 2) according to D &K: (8, 2) is neither kind nor unkind in latter game implying no rejections of (8, 2) in $\Gamma _{11}$, but some rejections of this offer in $\Gamma _{8}$.

Summarizing our predictions, we rank ($\Psi$) our games from those with the least likely rejection to the most likely rejection of (8, 2) according to D &K and F &F in Table 2 (i.e., games with a lower $\Psi$ are predicted to have less rejections than games with a higher $\Psi$). Of course, this means on the within-subject level that responders who reject a game with a low $\Psi$ are predicted to reject all games with higher $\Psi$s as well. Notice that a rank of 0 results from D &K’s prediction that no responder should reject (8, 2) in this game. Finally, the ranks of $\Gamma _1$, $\Gamma _3$, $\Gamma _6$ and $\Gamma _7$ depend on the individual parameter $\epsilon _{R}$. As $1\ge \epsilon _{R}\ge 0$, $\epsilon _{R}\approx 0$ for some players implies the ranks of 1, 1, 2, and 3, while $\epsilon _{R}\approx 1$ implies the ranks of 4, 4, 4, 4, respectively. As we are facing in the experiment a random sample, it seems plausible to assume in the aggregate the ranks of 1, 1, 2, and 3, whereas this needs not be the case at the individual level.

**Table 2:** Payoff alternatives in $\Gamma _1$ to $\Gamma _{12}$ along the game’s rank according to the likelihood (least to most) of a rejection for (8, 2) according to the corresponding theories

	Green	Red	Yellow	Blue	$\Psi ^{D \& K}(8,2)$	$\Psi ^{F \& F}(8,2)$
$\Gamma _1$	(8, 2)	(8, 2)			0	1
$\Gamma _2$	(8, 2)	(5, 5)			3	4
$\Gamma _3$	(8, 2)	(9, 1)			0	1
$\Gamma _4$	(8, 2)	(7, 3)			1	4
$\Gamma _5$	(8, 2)	(4, 3)			1	4
$\Gamma _6$	(8, 2)	(3, 7)			4	2
$\Gamma _7$	(8, 2)	(3, 4)			2	3
$\Gamma _8$	(10, 0)	(9, 1)	(8, 2)	(5, 5)	1	4
$\Gamma _9$	(9, 1)	(7, 3)	(8, 2)	(5, 5)	2	4
$\Gamma _{10}$	(7, 3)	(6, 4)	(8, 2)	(5, 5)	3	4
$\Gamma _{11}$	(9, 1)	(10, 0)	(8, 2)	(6, 4)	0	4
$\Gamma _{12}$	(9, 1)	(7, 3)	(8, 2)	(6, 4)	1	4

### Setting

The experiment is conducted as an online survey. Each participant plays every game in the role of either the responder, or the proposer. Subjects are randomly assigned to one of the two roles that they keep for the whole experiment. This allows us a within-subject analysis across games. On average, nine out of ten subjects participate as a responder, while approximately every tenth subject is assigned to be the proposer.^12^ For responders, we apply the strategy method, in which responders have to accept or reject every possible payoff allocation (Selten 1967). Consequently, each responder has to make 34 choices, each proposer 12 choices.

The experiment starts such that participants receive an invitation email including a link to access the online interface of the experiment. While accessing the interface, subjects are first familiarized with the procedure of the experiment and the instructions of the game on several pages on the screen. Participants are informed about all parameters of the game (including the payoff procedure) at this stage. Subsequently, participants submit all their choices without any feedback. The games are presented sequentially without the possibility to review earlier choices. The order of the games is randomized for each participant in order to exclude order effects. Payoffs in the experiment are denominated in Talers that we exchange at 1 Taler for 2 Euros at the end of the experiment. During the experiment, all participants have on all decision screens the option to open an extra window showing again the instructions of the game. Finally, all participants have to fill out a short socio-demographic questionnaire. After the survey is completed by all participants, payment is determined from one randomly drawn game for every tenth formed pair of players. Subjects are informed via email about their payoff and pick up their earnings at the office of the experimental laboratory of the University of Hamburg.

In total, 496 students (various fields) from the University of Hamburg participated in two waves between November 2013 and March 2014 (each wave ran several days). 52.6 % percent were female, the median age was 25 years. We used hroot for recruitment (Bock et al. 2014). The average length of the entire online survey was approximately 20 min including instruction time. From the 427 responders, we choose randomly 43, while we assigned 43 out of the 69 proposers to form pairs receiving payments. The payoffs are computed according to the responder’s decision corresponding to the particular choice of the proposer. Average payoff among the all players receiving payoffs was 8.66 Euro, implying an expected payoff of 0.87 Euro for each responder and 5.40 for each proposer.^13^

## Results

### Aggregate reciprocity

**Fig. 1:**
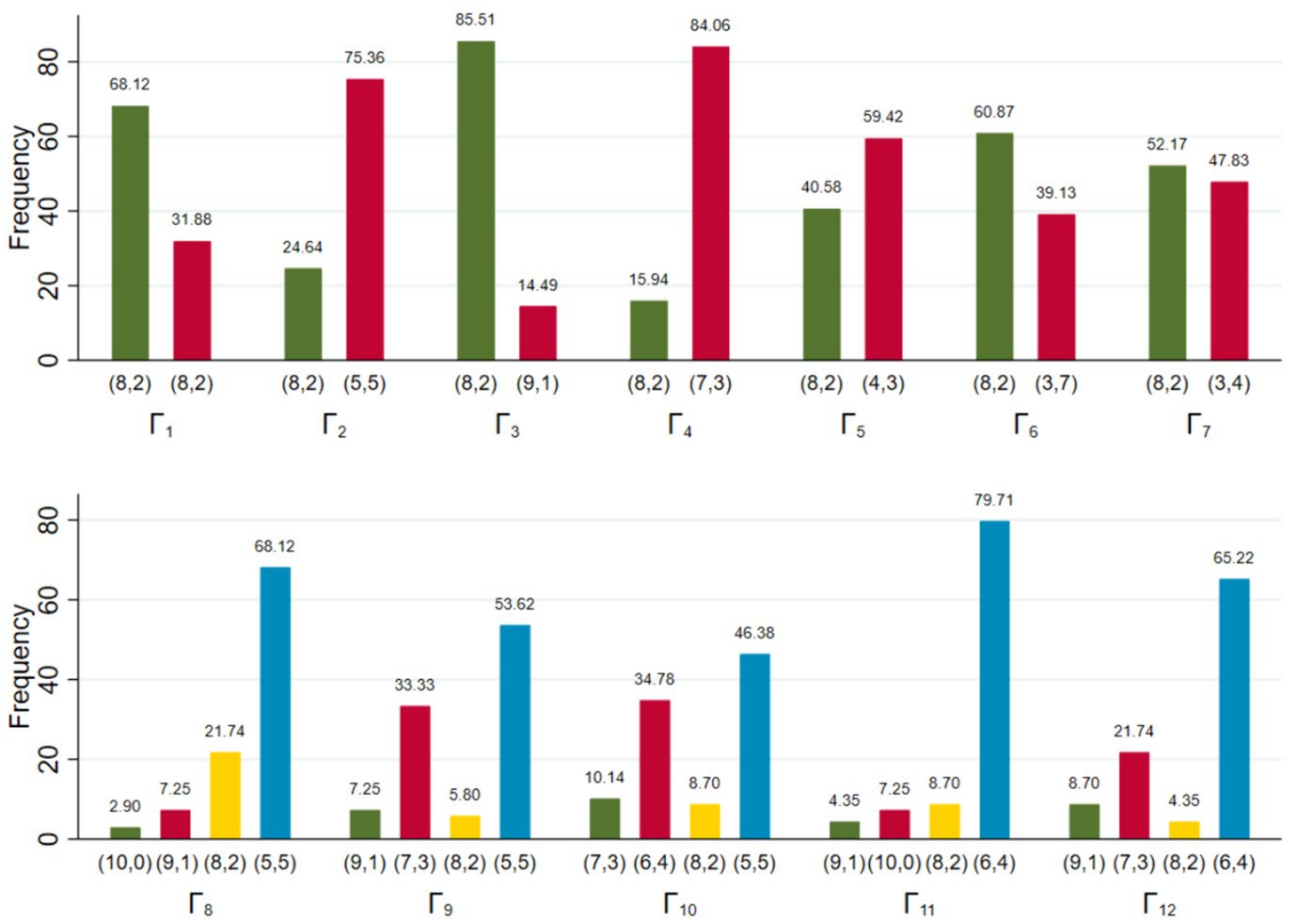
First mover decisions (in percent)

Let us start with proposers’ decisions. We have 69 of them in our sample.^14^ Fig. 1 illustrates first mover decisions for $\Gamma _{1}$ to $\Gamma _{12}$. In general, the majority of proposers behaves very kindly such that the majority chooses the kindest offer in all games (the only exceptions are $\Gamma _6$ and $\Gamma _7$). In $\Gamma _{3}$ (and, trivially, in $\Gamma _{1}$), this is (8, 2) (which is chosen by 86% of the proposers), while in the other simple games, (8, 2) is offered by 25% ($\Gamma _{2}$), 16% ($\Gamma _{4}$), 41% ($\Gamma _{5}$), 61% ($\Gamma _{6}$) and 52% ($\Gamma _{7}$) of the proposers. In the richer games, the choice of (8, 2) differs substantially over games: proposers offer (8, 2) in $\Gamma _{8}$ (22%) significantly more often than in $\Gamma _{9}$ (6%), in $\Gamma _{10}$ (9%), in $\Gamma _{11}$ (9%), and in $\Gamma _{12}$ (4%).^15^ Overall, it seems that (8, 2) is not the most popular, but not an irrelevant alternative in all games.

**Fig. 2:**
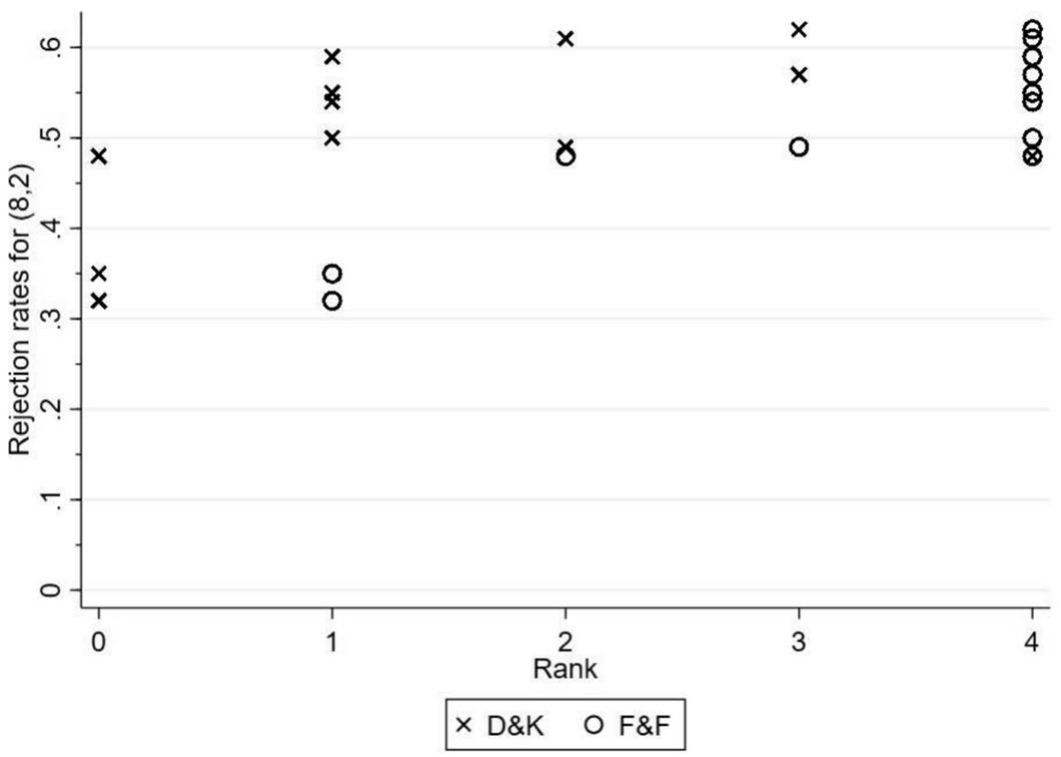
The y-axis displays the rejection rates for (8, 2) in $\Gamma _1$ to $\Gamma _{12}$. On the x-axis, the respective rank is plotted according to $D \& K$ and $F \& F$ with 0 reflecting the lowest and 4 the highest likelihood for rejection

Now, let us turn to the responders’ decisions. Table 3 and Fig. 2 report the rejection rate of (8, 2) in games $\Gamma _1$ to $\Gamma _{12}$.^16^ As expected, non-reciprocal social preferences fail to characterize responders’ decisions correctly. That is, neither are rejection rates similar across all games nor across the sub-sample $\Gamma _1, \Gamma _2$, $\Gamma _4$, $\Gamma _5$, $\Gamma _6$ $\Gamma _7$, and $\Gamma _{10}$. For instance, the rejection rates of $\Gamma _2$ and $\Gamma _6$, $\Gamma _2$ and $\Gamma _{10}$, and $\Gamma _4$ and $\Gamma _7$ are significantly different ($p<0.002$).^17^

With regard to reciprocal preferences, there are some observations in line with both models for games $\Gamma _1$ to $\Gamma _5$. That is, out of 427 subjects, 235 reject (8, 2) in $\Gamma _4$, and 231 subjects in $\Gamma _5$, so that – in line with both models – we cannot reject the hypothesis that there are different rejection rates for green in $\Gamma _4$ and $\Gamma _5$ ($p=0.61$). However, contradicting Hyp_D&K_, 137 responders reject (8, 2) in $\Gamma _3$. Furthermore, the rejection rate for $\Gamma _2$ (245 responders) is insignificantly different from the rate in $\Gamma _4$ ($p=0.17$), and only weakly significantly different from the rate in $\Gamma _5$ ($p=0.08$). Similarly, the rejection rates for $\Gamma _6$ (204 responders) and $\Gamma _7$ (209 responders) do not increase, but decrease significantly in comparison to $\Gamma _4$ ($p\le 0.002$) and to $\Gamma _5$ ($p\le 0.003$).

The results in $\Gamma _4$ and $\Gamma _5$ are in line with Hyp_F&F_. Yet, F &F fail to describe rejections in games with limited intention factors. That is, concerning the rejection rates in $\Gamma _6$ and $\Gamma _7$, we expect a smaller number in the first than in the second game (although there are some limitations to this expectations if $\epsilon _{R}$ is close to one for the majority of players; see our earlier comment at the end of Section 3.1). In contrast, there is no significant difference between the rates for both games ($p=0.59$). This result holds even if we restrict our focus on those responders who did not reject in $\Gamma _1$ (i.e., subjects without pronounced outcome concerns). Out of 267 responders who accepted both offers in $\Gamma _1$, 71 (70) rejected (8, 2) in $\Gamma _6$ ($\Gamma _7$); there is no significant difference between the two rejection rates ($p=0.8$). Likewise, the rejection rates of $\Gamma _1$ (152 responders) and $\Gamma _3$ differ weakly significantly ($p=0.08$) despite the predictions of Hyp_F&F_.

Nonetheless, our overall results suggest that F &F characterize behavior more accurately. That is, rejection rates in the simple games follow the predictions of F &F – particularly for fully intentional decisions, while they are poorly described by D &K.

Next, we look at the rejection rate of (8, 2) in $\Gamma _8$ through $\Gamma _{12}$. We observe two “blocks” of games with respect to their rejection rates. $\Gamma _9$, $\Gamma _{10}$ and $\Gamma _{12}$ have all rejection rates of approximately 0.6, while $\Gamma _{8}$ and $\Gamma _{11}$ have rejection rates of approximately 0.5. All rejection rates in the first block (260/263/251) are significantly higher than rejection rates in the second block (214/205; $p<0.001$), while within blocks, there is only one weakly significant difference between $\Gamma _{10}$ and $\Gamma _{12}$ ($p=0.08$, all other comparisons $p\ge 0.18$). Hence, contradicting Hyp_D&K_, there is little evidence that the sequence of rising distances to the reference value triggers rejections in a systematic way, whereas the same distance to the reference value leads to significantly different rejection rates between $\Gamma _8$ and $\Gamma _{12}$ ($p<0.001$). Likewise, a comparison across simple and richer games show significant different rejection rates between $\Gamma _1$ and $\Gamma _{11}$ ($p<0.001$).

Similarly, predictions according to Hyp_F&F_ are misaligned with results due to the lower rejections rate of (8, 2) in $\Gamma _8$ and $\Gamma _{11}$ compared to $\Gamma _9$, $\Gamma _{10}$ and $\Gamma _{12}$. In contrast to our earlier results, which corroborate with F &F’s model, we provide here evidence for a lack of generality (or a limiting specificity) of F &F’s model. More precisely, by changing the structure of the games in a way that we add a number of alternatives, reciprocal behavior cannot be satisfactorily described by F &F’s intention-based model anymore. Interestingly, the comparison across simple and richer games also casts some doubts onto F &F’s model (e.g., $\Gamma _4$ and $\Gamma _{12}$: $p=0.04$; or $\Gamma _2$ and $\Gamma _{8}$: $p<0.001$), although in those cases the alternatives of the simple games are subsets of the alternatives in the richer games. We discuss this point in greater detail in the Section 5.

Provided our observations, it seems that neither D &K’s model nor F &F’s model characterize rejection behavior in the richer games accurately. Particularly, both models fail to predict the occurrence of the two blocks. The results suggest that both models miss to incorporate an important characteristic of reciprocity.

**Table 3:** The first column reports observed rejection rates for (8, 2) in ascending order for $\Gamma _1$ to $\Gamma _{12}$

Game$\{ \textrm{Alternatives} \}$	Rejection rates for 8, 2	$\Psi ^{D \& K}(8,2)$	$\Psi ^{F \& F}(8,2)$	$\vartheta _P^{CA}(8,2)$	$\Psi ^{CA}(8,2)$
$\Gamma _3\{(9,1)\}$	0.32	0	1	$\epsilon _{R}$	1
$\Gamma _1\{(8,2)\}$	0.35	0	1	$\epsilon _{R}$	1
$\Gamma _{11}\{(9,1),(10,0),(6,4)\}$	0.48	0	4	$\epsilon _{R}$	1
$\Gamma _6\{(3,7)\}$	0.48	4	2	$\sigma _{R}$	2
$\Gamma _7\{(3,4)\}$	0.49	2	3	$\sigma _{R}$	2
$\Gamma _8\{(10,0),(9,1),(5,5)\}$	0.50	1	4	$\epsilon _{R}$	1
$\Gamma _5\{(4,3)\}$	0.54	1	4	1	3
$\Gamma _4\{(7,3)\}$	0.55	1	4	1	3
$\Gamma _2\{(5,5)\}$	0.57	3	4	1	3
$\Gamma _{12}\{(9,1),(7,3),(6,4)\}$	0.59	1	4	1	3
$\Gamma _9\{(9,1),(7,3),(5,5)\}$	0.61	2	4	1	3
$\Gamma _{10}\{(7,3),(6,4),(5,5)\}$	0.62	3	4	1	3

The second and and third column display the rank of rejection according to $D \& K$ and $F \& F$ with 0 reflecting the lowest and 4 the highest likelihood for rejection along the game’s values and ranks according to the modification of $\vartheta _{P}^{CA}(8,2)$ (for this see below)

### Individual reciprocity

In the following, we test for the consistency of our results on an individual level. For this, we test the personal implications of both models across games. In a first step, we test whether pairwise comparison of acceptances and rejections of two games leads to consistent results. That is, provided the decision in one game, the same subject has to make the same decision in another game associated to the same rank. Moreover, if the subject rejects (accepts) an offer, other offers associated with higher (lower) ranks ought to be rejected (accepted) by the same subject as well. Therefore, we will test the continuity of the two rankings across all games in a second step. That is, we will test whether the rejection of an offer implies the rejection of all games associated to a higher rank. Likewise, the acceptance of a game implies the acceptance of all games with a lower rank.

Let us start with the first step and the pairwise comparison of simple games according to D &K. In line with Hyp_D&K_, we cannot reject the hypothesis that the same subjects reject $\Gamma _4$ and $\Gamma _5$ ($p=.61$).^18^ At the same time, we have to reject the hypothesis that at least all responders rejecting (8, 2) in $\Gamma _4$ and $\Gamma _5$ (203 responders do so) reject (8, 2) in $\Gamma _2$, $\Gamma _6$ and $\Gamma _7$ as well ($p<0.001$): only 151 responders reject (8, 2) in all five games. Likewise, the prediction that responders rejecting (8, 2) in $\Gamma _7$ reject (8, 2) in $\Gamma _2$ and $\Gamma _6$ is not supported by the data ($p<0.001$): only 160 responders reject (8, 2) in all three games. Finally, the prediction that responders who reject (8, 2) in $\Gamma _2$ do so in $\Gamma _6$ as well is also not supported by the data ($p<0.001$): 186 responders reject (8, 2) in both games.

Turning to F &F, we find that 107 responders reject (8, 2) in $\Gamma _1$ and $\Gamma _3$ (out of 152/137 rejecting $\Gamma _1/\Gamma _3$), so that there is weakly significant evidence that not the same subjects reject this offer in both games ($p=0.08$). Similarly, Hyp_F&F_ is not supported in the sense that from 204 (209) responders rejecting (8, 2) in $\Gamma _6$ ($\Gamma _7$), 153 (180) reject the same offer in $\Gamma _4$, $\Gamma _5$ and $\Gamma _7$ ($\Gamma _4$ and $\Gamma _5$). Here, we have to reject the hypothesis that the same subjects reject this offer in all four games ($p<0.001$, and $p=0.002$, respectively). However, we cannot discard the hypothesis that the same subjects reject (8, 2) in $\Gamma _4$ and $\Gamma _5$ ($p=0.61$): from 235 (231) rejecting (8, 2) in $\Gamma _4$ ($\Gamma _5$), 203 reject the offer in both games. In other words, F &F organizes the data well, if offers are fully intentional referring to their model.

For the richer games, D &K predict that responders rejecting (8, 2) in $\Gamma _8$ ($\Gamma _9$) are expected to reject the same offer in $\Gamma _9$ and $\Gamma _{10}$ ($\Gamma _{10}$). There is little evidence for the claims: from 214 (260) responders rejecting (8, 2) in $\Gamma _8$ ($\Gamma _9$), 194 (236) responders do so in $\Gamma _9$ and $\Gamma _{10}$ ($\Gamma _{10}$) as well ($p<0.001$ for both comparisons). Likewise, not the same responders reject (8, 2) in $\Gamma _4$, $\Gamma _5$, $\Gamma _8$ and $\Gamma _{12}$ ($p<0.001$), $\Gamma _{7}$ and $\Gamma _9$ ($p<0.001$), and $\Gamma _2$ and $\Gamma _{10}$ ($p=0.01$). Finally, from 167 responders rejecting (8, 2) in $\Gamma _4$, $\Gamma _5$, $\Gamma _8$ and $\Gamma _{12}$, 137 reject (8, 2) in $\Gamma _7$, $\Gamma _9$, $\Gamma _2$, $\Gamma _{10}$ and $\Gamma _6$ as well. Again, the claim that the same responders reject (8, 2) across all those games is not supported ($p<0.001$).

We continue our analysis by testing F &F in the context of richer games: according to Hyp_F&F_, the same responders reject (8, 2) in games $\Gamma _2$, $\Gamma _4$, $\Gamma _5$ and $\Gamma _8$ to $\Gamma _{12}$. This claim is not supported by the data ($p<0.001$). Even if we restrict our analysis to games $\Gamma _8$ to $\Gamma _{12}$, there is little evidence that the same responders reject (8, 2) in those games ($p<0.001$). However, we cannot reject the claim that the same responders reject (8, 2) in $\Gamma _9$, $\Gamma _{10}$ and $\Gamma _{12}$ ($p=0.19$). Thus there seems to be an important difference between $\Gamma _9$, $\Gamma _{10}$ and $\Gamma _{12}$ on the one hand, and $\Gamma _8$ and $\Gamma _{11}$ on the other that influences reciprocity substantially in our experiment.

Now, we turn to the second step of the analysis: we check the continuity of the two rankings across all games. That is, provided that a subject rejects a game, she rejects all games with the same and lower ranks as well. Likewise, provided that a subject accepts a game, she accepts all games with the same and higher ranks as well. For instance, suppose a subject accepts the offer (8, 2) in $\Gamma _{7}$. Since $\Gamma _{7}$ has the rank 2 according to D &K, the subject ought to accept all offers in games with higher or equal ranks, too (i.e., $\Gamma _1,\Gamma _3,\Gamma _4,\Gamma _5,\Gamma _8,\Gamma _9,\Gamma _{11}$, and $\Gamma _{12}$). In turn, rejecting (8, 2) in $\Gamma _{7}$ (rank 3 referring to F &F), a subject ought to reject all offers in games with lower or equal rank 3 according to F &F (i.e.,$\Gamma _2,\Gamma _4,\Gamma _5,\Gamma _8,\Gamma _9,\Gamma _{10},\Gamma _{11}$, and $\Gamma _{12}$).

**Fig. 3:**
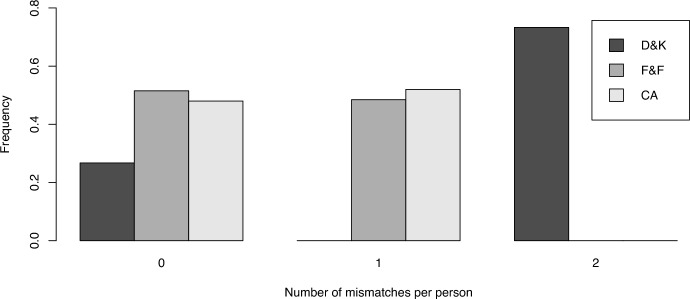
Frequency of individual deviations from the prediction patterns according to the theories (for ‘CA’ see below)

Altogether, the prediction of both D &K and F &F leads to five patterns for acceptances and rejections each.^19^ We check for each subject whether the subject’s acceptances and rejections match one of the patterns and, if not, the minimum mismatches from one of the patterns both for D &K and F &F (and CA, see below). Figure 3 illustrates the frequency of subjects whose acceptances and rejections of (8, 2) match exactly one predicted pattern (denoted as ‘0’), match a predicted pattern except one or two choices at most (‘1’ and ‘2’), respectively (two out of twelve choices is the maximum number of individual mismatches). Overall, only 114 subjects (out of 427) show no mismatch according to the predictions from D &K, while 220 according to the predictions from F &F. Thus, the former model yields significantly fewer no-mismatches than the latter ($p<0.001$, two-sided proportion test comparing both numbers). In line with this observation, we find an average number of 1.47 mismatches for D &K and 0.48 mismatches for F &F. Notice that a large proportion of individual mismatches with D &K’s predictions results from rejections in $\Gamma _1,\Gamma _3$, and $\Gamma _{11}$ (i.e., D &K predict rank zero for those games). Thus, an advantage of the F &F model and a disadvantage of the D &K model is that the former (latter) model can (cannot) rationalize rejections based on inequity considerations.

## Discussion

Our results indicate for both models weaknesses when predicting perceived kindness. D &K provide with their $\kappa _{PR}^{D \& K}(a_P)$ a partition in the degree of kindness which is too detailed. F &F’s kindness measurement yields a partition of kindness which is too general in richer settings due to the pairwise comparison of offers to the extreme alternatives. Consequently, neither model can describe behavior fully. Therefore, we want to discuss a potential combination of both reciprocity models (‘Combined approach’, abbreviated ‘CA’). However, we do not attempt to present a fully elaborated model, but we want to sketch a possible avenue for the further development of research on reciprocity. On the one hand, we want to adjust F &F’s model so that it yields more predictive power in richer environments while keeping the number of mismatches as low as possible (though additional predictive power will lead almost inevitable to more mismatches). On the other hand, we want to incorporate the assessment of alternatives’ kindness according to the reference value approach to provide a sufficiently simple partition of kindness.

As our data corroborate in simple games F &F’s distinction between five generic cases of intention (and the distinction between the intention and the extent to which this alternative is considered to be kind or unkind), we do not seek to modify this feature.^20^ Hence, we propose a $\kappa _{PR}^{CA}(a_P)$ which consists of the product of an intention factor and the outcome term $\Delta _P(a_P)$ according to equation (6). However, the major extension is an assessment of *P*’s alternatives according to a reference value. Not only do our experimental results support this approach, but we consider it as unrealistic that in more complex situations – and we study in our experiment complexity only to the extent that we test games with four instead of two alternatives – the existence of one clearly (un)kind action determines the choice of another alternative as a fully intentional act of (un)kindness.

Thus, similar to D &K’s approach, we model intention relative to some reference value. Specifically, the reference value we propose, the median offer (i.e., the “middle” payoff within the set of corresponding end notes resulting from the consecutive choice of efficient strategies) provides the additional benefit of being robust against outliers in the set of alternatives (D &K discuss the problem of outlying payoffs extensively in their paper). Formally, let us denote with $\pi _i^{M}$ *i*’s median payoff among the set of payoffs resulting from *j*’s choice.^21^ Then, we define the intention factor of *P*’s move $\vartheta _{P}^{CA}(a_P)$ of offering a specific payoff combination $\pi _P^0,\pi _R^0$ as follows:

8 $$\begin{aligned} \vartheta _{P}^{CA}(a_P)= {\left\{ \begin{array}{ll} 1 &{} \text {if }\pi _R^0>\pi _R^M \text { and } \pi _R^0>\pi _P^0,\\ \epsilon _{R} &{} \text {if }\pi _R^0\le \pi _R^M \text { and } \pi _R^0\ge \pi _P^0,\\ \epsilon _{R} &{} \text {if }\pi _R^0\ge \pi _R^M \text { and } \pi _R^0\le \pi _P^0,\\ 1 &{} \text {if }\pi _R^0<\pi _R^M, \pi _R^0<\pi _P^0, \text { and }\exists {\tilde{\pi }}_{P} \in {\tilde{\Pi }}_P: {\tilde{\pi }}_P>\pi _R^M\\ \sigma _{R} &{} \text {if }\pi _R^0<\pi _R^M, \pi _R^0<\pi _P^0, \text { and }\forall {\tilde{\pi }}_{P} \in {\tilde{\Pi }}_P: {\tilde{\pi }}_P\le \pi _R^M, \end{array}\right. } \end{aligned}$$

where ${\tilde{\Pi }}_P$ be the set of proposer’s payoffs resulting from the acceptance of an alternative offer, ${\tilde{\pi }}_{P}$ be one element in ${\tilde{\Pi }}_P$, and $\sigma _{R}$ be another individual parameter with $0\le \epsilon _R \le \sigma _R \le 1$.

That is, like F &F’s approach, our intention factor differentiates between five categories of *R*’s outcomes resulting from *P*’s action: payoffs implying smaller payoffs for *P* than for *R* are considered as fully intentionally kind if they are larger than the reference value, whereas they are accidentally kind if they are smaller or equal to the reference value. In turn, there are accidentally unkind offers which imply larger payoffs for *P* than for *R* if they are larger or equal to the reference value. Finally, *P*’s action leading to *R*’s payoff being smaller than *R*’s reference value and *P*’s payoff is considered to be fully intentional unkind only if *P* could choose better alternatives for herself. That is, if the unkind offer is “somehow understandable” in the sense that all other alternatives yield less for *P* than *R*’s reference value, the offer is still perceived as intentionally unkind but not that much. Only, if there is at least one other alternative which yields for *P* more than *R*’s reference value, choosing the specific alternative is fully intentional (and unkind).

Notice that the latter two cases translate F &F’s observation that “the perception of the unfair offer depends on how much *j* has to sacrifice in order to make the more friendly offer” (F &F, 2006, p. 297) into the context of a global assessment of *i*’s payoffs.^22^ That is, if making a more friendly offer than (8, 2) implies that *P* earns less than *R*’s reference value, this offer is still unkind, but with limited intention.

Based on our reformulation of $\vartheta _{P}^{CA}(a_P)$, we obtain a new rank order for the likelihood of a rejection for (8, 2) which is reported in the last column of Table 3: the predictions based on $\vartheta _{P}^{CA}(a_P)$ follow qualitatively the one based on $\vartheta _{P}^{F \& F}(a_P)$ for the simple games. However, using $\vartheta _{P}^{CA}(a_P)$ one can predict the two blocks of rejection rates in the richer games. In addition, the rejection rates for (7, 3) in $\Gamma _9$, $\Gamma _{10}$ and $\Gamma _{12}$ follow the predicted pattern: in $\Gamma _9$ and $\Gamma _{12}$ where (7, 3) is unfavorable but mildly unkind, 101 and 106 responders reject the offer, while 151 do so in $\Gamma _{10}$ where this offer is fully intentionally unkind.^23^

Comparing the number of individual mismatches between decisions and predictions between both models, we obtain a similar performance of the combined approach and the F &F model (see our Fig. 3): for 48% of all subjects we compute no mismatch based on the prediction of the combined approach, whereas 52% based on the prediction of the F &F model. The difference between both frequencies is not significant ($p=0.338$, two-sided proportion test comparing both numbers). Hence it seems that the combined approach is able to sharpen the predictive power while keeping the good performance with regards to the low number of mismatches.

Finally, we run a series of linear probability regressions on the individual decisions whether to accept or reject the offer (8, 2) (reported in Table 4). We control for individual characteristics by including individual dummy variables for all subjects. Independent variables are the ranks resulting from our three models F &F, D &K and CA. We obtain remarkably high $R^2$ values in all regressions along constantly significant coefficients for all three ranks. Most importantly, we assess whether including the ranks of the Combined approach adds to the predictive power of the regressions by running likelihood-ratio tests. Results indicate that in all cases the explanatory power of the regressions increase significantly by adding the ranks of the Combined approach. That is, adding the new ranks to the ranks of the F &F model (i.e., (1) vs. (5)) and to the ranks of the D &K model (i.e., (2) vs. (6)), as well as to a combination of both models (i.e., (4) vs. (7)) contributes significantly to the explanation of variance in the rejections of (8, 2).

**Table 4:** Linear probability regressions

Dependent variable	Rejecting the offer (8, 2)						
	(1)	(2)	(3)	(4)	(5)	(6)	(7)
$\Psi ^{F \& F}$	0.068^∗∗∗^			0.062^∗∗∗^	0.046^∗∗∗^		0.049^∗∗∗^
	(0.004)			(0.004)	(0.005)		(0.005)
$\Psi ^{D \& K}$		0.037^∗∗∗^		0.025^∗∗∗^		0.009^∗∗^	0.015^∗∗∗^
		(0.003)		(0.003)		(0.004)	(0.004)
$\Psi ^{CA}$			0.083^∗∗∗^		0.047^∗∗∗^	0.077^∗∗∗^	0.034^∗∗∗^
			(0.005)		(0.006)	(0.006)	(0.007)
Constant	$-$0.220^∗∗^	$-$0.055	$-$0.180^∗∗^	$-$0.241^∗∗∗^	$-$0.252^∗∗∗^	$-$0.179^∗∗^	$-$0.255^∗∗∗^
	(0.088)	(0.090)	(0.089)	(0.088)	(0.088)	(0.089)	(0.088)
Observations	5124	5124	5124	5124	5124	5124	5124
Individual Dummies	Yes	Yes	Yes	Yes	Yes	Yes	Yes
R^2^	0.662	0.645	0.659	0.666	0.666	0.659	0.667
F-statistic	21.50***	20.01***	21.24***	21.82***	21.89***	21.22***	21.94***
LR-test: $\chi ^2(1)$					70.78***	204.90***	27.22***
					(1)vs.(5)	(2)vs.(6)	(4)vs.(7)

Significance levels *p<0.1, **p<0.05 and ***p<0.01

Overall, it seems that $\kappa _{PR}^{CA}(a_P)$ provides a good combination between F &F’s idea of a kindness term which differentiates between the intention of an action and the extend of kindness with D &K’s approach to assess an entire game by means of a reference value. This results in more diverse predictions particularly in the context of richer decision environments. For instance, in an ultimatum-type game with several alternatives, the assumption that players evaluate the kindness of a specific offer by pairwise comparisons between alternatives seems unrealistic to us at least due to the shire computational effort it takes to compare alternatives against each other. Rather, we follow D &K’s idea that players condense alternatives by means of reference values. As such, our approach is located in some sense half way between the models by D &K and F &F: it processes more information than the model by D &K. On the other hand, our approach generalizes over alternatives by a larger extent than F &F by forming reference values. Whether our modifications optimize the tradeoff between the generalisability of the model to various situations and the accurate prediction of specific behavior is an open question and requires future research.

## Conclusion

Although reciprocity is one fundamental cornerstone of human behavior, modeling reciprocity still is a challenge for social scientists. The current study analyzes two of the most established approaches, the reference value model by D &K and the intention factor model by F &F. We point out that there are two major differences between the two approaches: the first model measures perceived kindness of an action in relation to a reference value while the second model distinguishes between the intention of an action and the extent to which the action is perceived as being unkind or kind. The latter element of F &F’s model relies on the inequity of the proposed payoffs whereas the former element results from a pairwise comparison between alternatives.

We test elements of both models within the context of mini-ultimatum games with two and four alternatives. Results show that F &F’s approach works fine in the games with two alternatives, but has important drawbacks in the games with four alternatives, both with respect to the average numbers but also once we run a within-subject analysis. On the other hand, D &K’s model fails to characterize behavior within both contexts. However, we have to admit that we do not test D &K’s complete model excluding some equilibria based on mutual meanness. This may impair its predictive success in our setting. Despite the shortcoming, we conclude that D &K’s idea to measure perceived kindness in one variable, the distance to the equitable payoff, does not sufficiently capture the nature of perceived (un)kindness. Particularly for games with four alternatives, it seems that the pairwise comparison of alternatives does not predict behavior accurately. Therefore, we present and discuss a potential modification of F &F’s reciprocity model which includes elements of D &K’s approach. Testing the Combined approach’s predictions with our experimental data yields an appropriately low number of mismatches between subjects’ decisions and the predictions. Also the results of the regression analyses indicate a significant improvement of predictive power added by the Combined approach to the existing models.

To conclude, more research is needed to model reciprocity in a sufficient way. Perhaps, the question is not whether there is a true model mapping reciprocity, but whether there is a model that adequately balances the need for generalisability across different games with a satisfactory good predictability of behavior within a specific environment. Elsewhere, it has been shown that reciprocity itself encompasses a number of different subtypes of social utility (e.g., Nicklisch and Wolff (2012)). Therefore, we have to ask ourselves whether we want to model the behavior in one specific game which may trigger one specific form of reciprocity, or whether we want to rely on a general model, which, however, has less predicting power in special situations. The answer to this question we cannot provide here. Therefore, we would like to invite future research to follow this avenue, or, maybe, prove it wrong.

## Data Availability

Instructions and the complete data of the experiment are available here: https://osf.io/tm98j/?view_only=dd6232fc938e4c28ad5a04b0c901d2bb.

## Appendix: Predictions according to outcome concerned social utility

### Inequity aversion

Provided inequity averse preferences, we have to claim that responders either accept or reject (8, 2) in all games $\Gamma _1$ to $\Gamma _{12}$. The reason for this is rather obvious: as the same offer (8, 2) is considered throughout all games and inequity aversion preferences take only the outcome of a proposal into consideration, there is no difference in the utility resulting from acceptance across games, nor from rejection across games.

### Levine’s (1998) model

In the following we want to derive a prediction for behavior in our games according to Levine’s (1998) model. We choose this model, since it can be characterized as some intermediate step between models based on outcome concerns and reciprocity models. The reason for this is that the proposer partly reveals her taste for altruism through her choice among the alternatives of the game. This information updates the weight for altruism in the responder’s utility function and may lead to rejections if altruism is negative. Formally, we can define the utility function of player *i* (paired with *j*) according to Levine (1998) as:

$$\begin{aligned} U_{i}^{L} = \pi _{i}(a_i,a_j)+\frac{\alpha _i+\varrho _i \alpha _j}{1+\varrho _i}\pi _{j}(a_i,a_j) \end{aligned}$$

where $\pi _{i}(a_i,a_j)$ is *i*’s monetary payoff of an offer, while $\alpha _i$ is *i*’s taste for altruism ($-1<\alpha _i<1$); finally, $\varrho _i$ measures the importance of *j*’s altruism for *i*’s utility ($0\le \varrho _i\le 1$).

Suppose *i* is a proposer, while *j* is a responder. Of course, $\alpha _i$ and $\varrho _i$ are *i*’s private information. However, by choosing a specific offer in the game, the proposer *i* partly reveals her taste for altruism to the responder *j* who updates her utility $U_j^L$ accordingly. Therefore, *j*’s utility changes with *i*’s choice of alternatives in the game. Yet, it turns out that *i*’s choice of (8, 2) is uninformative in $\Gamma _1, \Gamma _2$, $\Gamma _4$, $\Gamma _5$, $\Gamma _6$, $\Gamma _7$, and $\Gamma _{10}$ in the sense that it only reveals $a_i<1$ which is known from the beginning. As an example, consider $\Gamma _2$. Here, choosing (8, 2) implies $8+\frac{\alpha _i+\varrho _i \alpha _j}{1+\varrho _i}2>5+\frac{\alpha _i+\varrho _i \alpha _j}{1+\varrho _i}5$ which is equivalent to $1+\varrho _i(1-\alpha _j)>\alpha _i$. It follows that the maximum of $\alpha _i$ is 1.

Thus the choice of a specific offer in our mini-ultimatum games does not result in an update of *j*’s belief concerning $a_i$. It follows that in all of the previously mentioned games *j* rejects the offer of (8, 2) if $0>2+\frac{\alpha _j+\varrho _j \alpha _i}{1+\varrho _j}8$. It follows that *j* rejects if $\alpha _j<{\hat{\alpha }}$ with ${\hat{\alpha }}\in (-1,\ldots ,0.5]$ depending on *j*’s specific $\varrho _j$. That is to say, if a responder rejects (8, 2) in one of the games $\Gamma _1, \Gamma _2$, $\Gamma _4$, $\Gamma _5$, $\Gamma _6$, $\Gamma _7$, or $\Gamma _{10}$, she should reject (8, 2) in all seven games. Following the same rational, Levine’s model predicts that *i* does not offer 8, 2 in $\Gamma _3$, $\Gamma _8$, $\Gamma _9$, $\Gamma _{11}$, and $\Gamma _{12}$. Therefore, we are hardly able to form predictions with respect to responder’s behavior.

Notice that our deliberations rely on the linear specification of Levine’s model. One may think of a more general interpretation of the model. Let us assume that we can use the frequency by which proposers choose (8, 2) as an indicator for the degree of unkindness of this offer. This leads then to an update of *R*’s belief regarding $\alpha _P$ and, consequently, *R*’s rejection rate: that is, the more frequent (8, 2) is chosen by the proposers, the less mean it is considered by the responders, and, therefore, rejected less frequently (e.g., Utikal and Fischbacher (2014) follow this idea). This leads, for instance, for the richer settings $\Gamma _8$ to $\Gamma _{12}$ to the prediction that the rejection rate for (8, 2) in $\Gamma _8$ is lower than in $\Gamma _9$ to $\Gamma _{12}$ (since (8, 2) is offered significantly more frequently in $\Gamma _8$ than in $\Gamma _9$, $\Gamma _{10}$, $\Gamma _{11}$, and $\Gamma _{12}$, see our proposer analysis at the beginning of Section 4.1). Although not explaining our data fully – the prediction is violated by comparing $\Gamma _8$ to $\Gamma _{11}$ ($p=0.584$, two-sided proportion test comparing both rejection rates) – we would like to invite researchers to consider this often overlooked model for their future studies.

### Predicted acceptance and rejection patterns according to the three approaches

See Table 5.

**Table 5:** Choice patterns for (8, 2) in $\Gamma _1$ to $\Gamma _{12}$ according to the three approaches; ‘a’ indicates predicted acceptances, ‘r’ predicted rejections

Game	D &K pattern					F &F pattern					CA pattern			
	(I)	(II)	(III)	(IV)	(V)	(I)	(II)	(III)	(IV)	(V)	(I)	(II)	(III)	(IV)
$\Gamma _1$	a	a	a	a	a	a	a	a	a	r	a	a	a	r
$\Gamma _2$	a	a	r	r	r	a	r	r	r	r	a	r	r	r
$\Gamma _3$	a	a	a	a	a	a	a	a	a	r	a	a	a	r
$\Gamma _4$	a	a	a	a	r	a	r	r	r	r	a	r	r	r
$\Gamma _5$	a	a	a	a	r	a	r	r	r	r	a	r	r	r
$\Gamma _6$	a	r	r	r	r	a	a	a	r	r	a	a	r	r
$\Gamma _7$	a	a	a	r	r	a	a	r	r	r	a	a	r	r
$\Gamma _8$	a	a	a	a	r	a	r	r	r	r	a	a	a	r
$\Gamma _9$	a	a	a	r	r	a	r	r	r	r	a	r	r	r
$\Gamma _{10}$	a	a	r	r	r	a	r	r	r	r	a	r	r	r
$\Gamma _{11}$	a	a	a	a	a	a	r	r	r	r	a	a	a	r
$\Gamma _{12}$	a	a	a	a	r	a	r	r	r	r	a	r	r	r

## Footnotes

1 Alternative approaches such as distributional concerns (e.g., Fehr and Schmidt (1999)) or guilt-aversion (e.g., Battigalli and Dufwenberg (2007)) have been shown to explain pro-social behavior partly, but not comprehensively (e.g., Andreoni et al. (2003); Nicklisch and Wolff (2012)).

2 In February 2024, the paper by Falk & Fischbacher is cited more than 3920 times, while Dufwenberg & Kirchsteiger is cited more than 2690 times according to Google Scholar.

3 Other models incorporating reciprocity follow the same logic, but apply slightly different techniques (e.g., Cox et al. (2007)).

4 We clarify the meaning of “normally” below.

5 Notice that we do not test the complete approach by D &K. We dismiss some equilibria based on mutual meanness as not plausible in our setting. For further details see Section 2.

6 Of course, both approaches cover much richer settings. Here, we restrict our deliberations to the experimental setting for the notational simplicity.

7 For this argumentation, “very” refers to cases in which the immaterial utility component outweighs the material utility component for both players.

8 Notice that the described behavior creates a logical twist for reciprocal preferences. It appears questionable whether a mutual mean action allowing players to maximize the reciprocal utility is not in fact nice: it creates a higher utility than the one resulting from the acceptance of a small offer. If so, it turns the starting point of this thought experiment – rejecting offers is mean – absurd.

9 If the proposer seeks rejections, this response may not be considered to be mean any longer, while accepting a mean offer creates an equally bad option for the responder. We guess that the coordination on such an unusual equilibrium appears very difficult in an anonymous experimental setting and may be desirable only for a very small number of players. Similar difficulties with distributional fairness preferences (e.g., inequality aversion) are excluded by the assumption that players do not throw away money for the sake of a reduction of income equalities (see, e.g., Fehr and Schmidt (1999), p. 824).

10 In their reference value model, Dufwenberg and Kirchsteiger (2004) refer to it as a “sequential reciprocity equilibrium.”

11 Notice that the total sum of Talers for alternative red in $\Gamma _5$ and $\Gamma _7$ yields less than ten. Therefore, one may argue that efficiency concerns could change the decisions in those games in a systematic way. As we will show in the result section, there are no indications that efficiency concerns influence decisions in $\Gamma _5$ and $\Gamma _7$, nor are the results of these games particularly important for the general picture of our results.

12 The lower probability for responders being selected follows a similar technique for random respondership applied by Grosskopf (2003) and Güth et al. (1997).

13 Expected (hourly) earnings correspond with previous experiments conducted via the internet or newspapers (e.g., Bosch-Domenech et al. (2002); Güth et al. (2003, 2007); Drehmann et al. (2005)).

14 Of course, their decisions are difficult to interpret as they are influenced by proposers’ fairness considerations, but also anticipated fairness needs of responders. Nonetheless, we report our data in order to provide a complete picture of our experiment.

15 $p=0.013$, $p=0.058$, $p=0.058$, and $p=0.005$, two-sided proportion tests, comparing frequencies of $\Gamma _{8}$ and $\Gamma _{9}$, $\Gamma _{8}$ and $\Gamma _{10}$, $\Gamma _{8}$ and $\Gamma _{11}$, and $\Gamma _{8}$ and $\Gamma _{12}$.

16 The empirical rejection rates for alternatives green and red are identical in $\Gamma _1$.

17 Throughout this subsection, we use a two-sided, paired t-test for the assessment of statistical significance.

18 Throughout this subsection, we use a two-sided Friedman test for the assessment of statistical significance.

19 For example, accepting (8, 2) in $\Gamma _1,\Gamma _3,\Gamma _4,\Gamma _5,\Gamma _7,\Gamma _8,\Gamma _9,\Gamma _{11}$, and $\Gamma _{12}$, while rejecting (8, 2) in $\Gamma _2,\Gamma _6$, and $\Gamma _{10}$ is one pattern predicted by D &K. Trivially, acceptance of (8, 2) in all games is another pattern predicted both by D &K and F &F, whereas rejecting (8, 2) in $\Gamma _1,\Gamma _3$, and $\Gamma _{11}$ cannot be rationalized by D &K. All predicted patterns are described in the Appendix.

20 On a side-note, we consider the fact that there is no significant difference of rejection rates within both blocks – although one game per block yields in sum less than 10 Talers – as evidence that efficiency concerns are less important in this setting.

21 We define $\pi _i^{M}$ implicitly with respect to the cumulative distribution function *F*(*x*) on *i*’s set of payoffs resulting from *i*’s and *j*’s choice of any combination of efficient strategies in a game: $\pi _i^{M}$ satisfies both inequalities $\int _{(-\infty ,\pi _i^{M}]}dF(x)\ge 0.5$ and $\int _{[\pi _i^{M},\infty )}dF(x)\ge 0.5$.

22 In this quote, *j* refers to the proposer and *i* to the responder in the context of our setting.

23 $p<0.001$ for the hypothesis that the rejection rate in $\Gamma _{10}$ equals one in the other two games, whereas $p=0.466$ for the hypothesis that the rejection rates in $\Gamma _9$ and $\Gamma _{12}$ are the same.
